# Histone demethylase inhibitor KDM5-C70 regulates metabolomic and lipidomic programming during an astrocyte differentiation of rat neural stem cell

**DOI:** 10.1038/s41598-025-88636-7

**Published:** 2025-02-13

**Authors:** Minki Shim, Thin Thin San, Bohyun Shin, Hyojeong Lee, Sang Beom Han, Dong-Kyu Lee, Hyun-Jung Kim

**Affiliations:** College of Pharmacy, Chung-Ang University, 84, Heukseok-ro, Dongjak-gu, Seoul, 06974 Republic of Korea

**Keywords:** Metabolomics, Lipidomics, Neural stem cell differentiation, KDM5-C70, Epigenetic modification, Phosphatidylethanolamine Biosynthesis, Mass spectrometry, Lipidomics, Metabolomics, Neural stem cells

## Abstract

Lysine-specific histone demethylase (KDM) 5 inhibition by KDM5-C70 induces astrocytogenesis and highlights the importance of modulation of histone methylation in cell fate specification. This study investigated the role of the histone demethylase inhibitor KDM5-C70 in modulating the metabolic and lipidomic landscape during astrocyte differentiation of rat neural stem cells (NSCs). Using chemical derivatisation combined with gas chromatography-mass spectrometry, 42 metabolites were detected, indicating potential regulation of phospholipid metabolism. Subsequent lipidomic analysis, employing reverse-phase liquid chromatography with high-resolution quadrupole time-of-flight mass spectrometry, identified 180 lipid species and 9 lipid subclasses. Integrative analysis revealed that KDM5-C70 promoted astrocytogenesis through epigenetic changes linked to the attenuation of phosphatidylethanolamine (PE) biosynthesis pathways. The reduced expression of transcripts related to PE highlighted the significance of the PE pathway in influencing cell fate decisions. These quantitative metabolomic and lipidomic analyses not only advance our understanding of NSC differentiation but also lay the groundwork for potential therapeutic strategies targeting metabolic pathways in neurodegenerative diseases and neural injuries.

**Supplementary Information:**

The online version contains supplementary material available at 10.1038/s41598-025-88636-7.

## Introduction

Neural stem cells (NSCs) are undifferentiated cells capable of self-renewal and differentiation into neurons, astrocytes, and oligodendrocytes^1,2^. Their differentiation potential and presence in the adult brain make them promising candidates for cell therapy^3^. Epigenetic mechanisms play crucial roles in cell fate decisions by regulating gene expression without altering DNA sequences^4,5^. These mechanisms impose stable gene expression patterns on differentiating cells^6^, allowing them to acquire distinct identities and functions despite having identical DNA sequences^7^. Key epigenetic mechanisms include DNA methylation, histone modification, and regulation by non-coding RNAs^8–10^. Histone modifications encompass acetylation and methylation^11,12^.

Many epigenetic studies have targeted histone lysine methylation and demethylation to regulate cell differentiation^13–15^. Methylation of histone (H)3 lysine (K)4, H3K36, or H3K79 activates gene transcription, whereas methylation of H3K9, H3K27, or H3K20 is associated with gene repression^16,17^. Histone methylation is regulated by methyltransferases and demethylases^18,19^, making histone demethylases important targets for controlling stem cell fate^13–15^. Lysine-specific histone demethylase 5B (KDM5B) is crucial for mouse embryonic stem cell differentiation along the neural lineage, correlating with the efficient silencing of stem and germ cell genes^20^. Our previous study on the effects of the KDM5 inhibitor KDM5-C70 on NSC fate control revealed enhanced astrocytogenesis and increased levels of H3K4me3 in the glial fibrillary acidic protein (GFAP) promoter of KDM5-C70-treated NSCs^14^. These findings suggest that KDM5 inhibition by KDM5-C70, which prevents the removal of methyl groups from H3K4, induces astrocytogenesis and highlights the importance of modulation of histone methylation in cell fate specification.

Metabolomics is a powerful tool for understanding biochemical mechanisms and provides insights into the underlying processes within cells^21–23^. Metabolism encompasses all biochemical reactions in a cell^24–26^, with metabolites acting as substrates and cofactors for epigenome-modifying enzymes^27^. This suggests that metabolites can directly influence or respond to the cellular demands of epigenetically modified programs^28^. Therefore, stem cell differentiation via epigenetic mechanisms likely involves alterations in metabolic signatures that play a significant role in cell fate changes^22,29^. Numerous metabolomics studies have shown that unique metabolic switching during stem cell differentiation regulates cellular homeostasis^30–32^. Cell lineage specification involves perturbing metabolic pathways to meet bioenergetic demands, cellular signalling, one-carbon metabolism (S-adenosylmethionine-associated), and changes in histone methylation^33^. Understanding intrinsic metabolic reprogramming during cell differentiation through epigenetic modifications is essential for sophisticated control of stem cells and their differentiation for medical and scientific purposes.

In this study, we aimed to examine the metabolic perturbations triggered by KDM5 inhibition using KDM5-C70 and found that metabolic reprogramming through epigenetic modifications plays a crucial role in NSC differentiation, particularly in astrocytogenesis. We employed integrative metabolomic and lipidomic profiling techniques, using gas chromatography (GC) and reverse-phase liquid chromatography (LC) coupled with mass spectrometry (MS), to investigate the effects of KDM5-C70 during NSC differentiation. This approach highlighted distinct metabolic and lipidomic perturbations within metabolic networks. We also explored the potential inhibition of enzymes related to gene expression during lipid metabolism following KDM5-C70 treatment. This study emphasizes the crucial role of phosphatidylethanolamine (PE), a key precursor of phospholipid biosynthesis, in the differentiation of NSCs into astrocytes.

## Methods and materials

### Chemicals

Water and methanol were obtained from J.T. Baker (Phillipsburg, NJ, USA). Chloroform, N,O-bis(trimethylsilyl) trifluoroacetamide with trimethylchlorosilane (BSTFA, 1% TMCS, 99%), pyridine, and methoxyamine hydrochloride were sourced from Sigma-Aldrich (St. Louis, MO, USA). KDM5-C70 was purchased from MedChemExpress (HY-120400; Monmouth Junction, NJ, USA), and dimethyl sulfoxide (DMSO) was obtained from Sigma-Aldrich (472301; St. Louis, MO, USA).

## NSC culture and astrocyte differentiation

All rat experiments were conducted following the guidelines of Chung-Ang University, the National Institutes of Health (NIH) for animal care, and were approved by the Animal Care Committee of Chung-Ang University (permission numbers: 13–0049 and 2014-00032). Embryonic day 14 (E14) Sprague-Dawley rats (Orient Bio Inc., Gyeonggi-do, Korea) were euthanised by transient CO_2_ exposure, followed by cervical dislocation. NSCs were isolated from the cortex of the embryos and cultured at a seeding density of 200,000 cells/mL in Dulbecco’s modified Eagle’s medium/F12 supplemented with 1% (v/v) antibiotic-antimycotic, 2% (v/v) B27 (15240-062; 17504-044; both from Thermo Fisher Scientific, Waltham, MA, USA), 20 ng/mL epidermal growth factor (EGF), and 20 ng/mL fibroblast growth factor 2 (FGF2) (GF144; GF003AF; Merck Millipore, Burlington, MA, USA). The cells were cultured as neurospheres for 6 days to allow for expansion under 5% CO_2_ at 37 °C, with the medium replenished every 2 days. Following expansion, neurospheres were dissociated using Accutase (SCR005; Chemicon, Temecula, CA, USA) and seeded as single NSCs onto cell culture plates pre-coated with 0.01% poly-D-lysine (P0899; Sigma-Aldrich, MO, USA) and 10 µg/mL laminin (23017-015; Invitrogen, Carlsbad, CA, USA). After 1 day of expansion, NSCs were treated with either 0.1% DMSO as a control vehicle or 25 µM KDM5-C70 without EGF and FGF2 to induce differentiation. Following the designated treatment duration, cells were harvested for further experimentation, as indicated in the figure legends. This study is reported following ARRIVE guidelines.

## Immunocytochemistry (ICC) and cell counting

The procedure for ICC analysis was as follows. The cells were fixed with 4% paraformaldehyde (SC-281692; USB Products, Cleveland, OH, USA) for 30 min. After fixation, cells were rinsed with phosphate-buffered saline (PBS) and blocked with 5% normal goat serum (S26; Merck Millipore, CA, USA) containing 0.2% Triton X-100 in PBS for 30 min. Primary antibodies targeting GFAP (rabbit anti-GFAP antibody, 1:500; Z0334, Agilent, Santa Clara, CA, USA) and  βIII Tubulin (TUBB)3 (mouse TuJ1 antibody, 1:1000; T5076, Sigma-Aldrich, MO, USA) were applied for 90 min. After incubation, cells were rinsed with PBS and treated with secondary antibodies conjugated to Cy3 (goat anti-rabbit IgG, 1:1000; 111-165-144; Jackson ImmunoResearch, West Grove, PA, USA) or Alexa Fluor 488 (goat anti-mouse IgG, 1:1000; A11001; Thermo Fisher Scientific, CA, USA) for 30 min, followed by rinsing with PBS. Nuclei were stained with DAPI (1:10,000, D9542, in PBS; Sigma-Aldrich, MO, USA) for 5 min. To avoid measurement bias, three random microscopic areas were selected for cell counting for each independent biological experiment. Fluorescence images were captured using an inverted fluorescence microscope (Leica, Hesse, Germany) and GFAP-, TuJ1-, or 4', 6-diamidino-2-phenylindole (DAPI)-positive cells were quantified. The percentage of GFAP- and TuJ1-positive cells was calculated by dividing their counts by the total number of DAPI-positive nuclei and multiplying by 100. The results are presented as fold-change compared to the DMSO-treated groups.

## Real-time reverse transcription polymerase chain reaction (RT-PCR)

Total RNA was extracted from the cell cultures using TRIzol reagent (15596026; Invitrogen, Carlsbad, CA, USA). Briefly, 1 µg of total RNA was reverse-transcribed into first-strand complementary DNA (cDNA) in a reaction volume of 20 µL using a QuantiTect Reverse Transcription Kit (Qiagen, Venlo, Limburg, Netherlands). Real-time RT-PCR was conducted to quantify the mRNA levels using iQTM SYBR Green Supermix (170-8882AP; Bio-Rad, Hercules, CA, USA). The primer sets for cDNA amplification were as follows: *Gfap*: 5′-agcggctctgagagagattc-3′ (forward) and 5′-agcaacgtctgtgaggtctg-3′ (reverse); *Tubb3*: 5′-agccctctacgacatctgct-3′ (forward) and 5′-attgagctgaccagggaatc-3′ (reverse); ethanolamine kinase (*Etnk)1*: 5′-cacctcagctctactgcacc-3′ (forward) and 5′-ctgacgagctatgagcctga-3′ (reverse); *Etnk2*: 5′-agcccccagctcttcaggtta-3′ (forward) and 5′-tctcatccttgaccagcgtg-3′ (reverse); phosphate cytidylytransferase 2, ethanolamine (*Pcyt2)*: 5′-gtgcgatggctgctatgac-3′ (forward) and 5′-ttggcaatctcctcgtcagt-3′ (reverse); selenoprotein I (*Selenoi)*: 5′-ttctatgcttcagcgccagg-3′ (forward) and 5′-aacagctcccctaatggggt-3′ (reverse); Phosphatidylserine decarboxylase (*Pisd)*: 5′-gctacgtgaaggtgtgctgt-3′ (forward) and 5′-acttttcggacccctgtgtt-3′ (reverse); phospholipase A and acyltransferase 3 (*Plaat3)*: 5′-tgggacctaaacaaaggcatcc-3′ (forward) and 5′-ccaacatagatggcccagtga-3′ (reverse); Glyceraldehyde 3-phosphate dehydrogenase (*Gapdh)*: 5′-agttcaacggcacagtcaag-3′ (forward) and 5′-gtggtgaagacgccagtaga-3′ (reverse). The qRT-PCR conditions were as follows: initial activation at 95 °C for 3 min, followed by 40 cycles of denaturation at 95 °C for 10 s, annealing at 58 °C for 15 s, and extension at 72 °C for 20 s. The housekeeping gene *gapdh* was used as an internal control.

## Polar metabolites and lipids extraction

Metabolites and lipids in differentiated cells were extracted using a modified freeze-thaw and biphasic extraction method, as previously described^34^. NSCs were treated with either 0.1% DMSO or 25 µM KDM5-C70 in the absence of EGF and FGF2 for 2 days. After the treatment period, the culture media were removed, and the cells were washed with ice-cold 1X PBS to eliminate any residual media. The cells were collected from the culture dish using a cell scraper and transferred to Eppendorf tubes. These tubes were centrifuged at 1000 rpm for 5 min at 4 °C, resulting in the formation of a cell pellet. The supernatant was carefully removed using a pipette (P200). Ice-cold autoclaved distilled water (ADW) was added to the tubes to wash the cell pellets, followed by centrifugation at 1000 rpm for 5 min at 4 °C. This washing step with ice-cold ADW was repeated twice to ensure thorough purification of the cell pellets. After the final centrifugation, the supernatant was aspirated, leaving purified cell pellets. The pellets were homogenised through three freeze/thaw cycles in liquid nitrogen. Following cell lysis, 500 µL of chloroform was added for protein precipitation and liquid-liquid extraction (LLE). Precipitated proteins were quantified using the Pierce BCA Protein Assay Kit (Thermo Fisher Scientific, Waltham, MA), according to the manufacturer’s protocol provided by the vendor. The liquid samples were vortexed for 1 min, centrifuged at 13,000 ×*g* for 5 min, and the non-polar layers were transferred to another microtube. LLE was repeated several times. The collected polar and non-polar layers were filtered through 0.45- and 0.20-µm syringe filters, respectively, and evaporated using a SpeedVac instrument.

### Polar metabolite profiling

The extracted polar metabolites were derivatised for GC-MS analysis using a previously described method^35^. For methoxyamination, 50 µL of a 20 mg/mL methoxyamine hydrochloride solution in pyridine was added to the dried polar metabolites and incubated for 90 min at 37 °C. Subsequently, for trimethylsilylation, 50 µL of N, O-Bis(trimethylsilyl) trifluoroacetamide with 1% trimethylchlorosilane solution was added to the sample and incubated for 1 h at 75 °C. The derivatised samples were transferred to glass vials and analysed using a Shimadzu QP2010 GC-MS system (Shimadzu Co. Kyoto, Japan). A DB-5 ms column (30 m × 0.25 mm i.d. × 0.25 μm film thickness) was used for chromatographic separation of polar metabolites. The injection temperature was set at 270 °C, and 1 µL of the sample was injected in the splitless mode. The column oven program was as follows: an initial temperature of 70 °C for 2 min, increased to 100 °C at 4 °C/min, held for 3 min, increased to 160 °C at 3 °C/min, held for 1 min, increased to 200 °C at 4 °C/min, held for 2 min, increased to 300 °C at 8 °C/min, and held for 8 min for a total of 68 min. Metabolites were identified based on their retention index (RI), which was calculated using an alkane mixture, and mass spectrum similarity to the NIST08 library. Quality control (QC) samples, composed of an equal volume from each sample, were analysed to ensure robustness during GC-MS analysis. QC sample data were collected between the acquisition of two samples. Metabolites identified with over 30% relative standard deviation (RSD) across the three QC samples were excluded from the dataset for multivariate statistical analysis. All GC-MS data were aligned using MetAlign software with parameters slightly modified from a previous study^36^.

## Lipid profiling

Dried lipids were reconstituted with 200 µL isopropanol (IPA) and analysed using a 1290 UPLC system equipped with a 6546 QTOF-MS (Agilent Technologies, Santa Clara, CA, USA). An ACQUITY UPLC BEH C18 column (2.1 × 100 mm, 1.7 μm, Waters, Milford, MA, USA) was used for lipid separation with a binary gradient elution at a flow rate of 0.15 mL/min at 40 °C. The mobile phases consisted of acetonitrile/water (2:8 v/v) as phase A and isopropanol/acetonitrile (6:4 v/v) as phase B, both containing 10 mM ammonium acetate and 0.1% acetic acid. The binary gradient was set as follows: 0.0 min B 40%, 3.0 min B 40%, 10.0 min B 85%, 23.0 min B 90%, 28.0 min B 100%, 30.0 min B 100%, 38.0 min B 100%, 40.0 min B 40%. The injection volume was 5 µL. Positive electrospray ionisation mode was used with specific chromatographic and spectrometric parameters for lipidomics. The detailed parameters were set as follows: sheath gas flow, 11 L/min; sheath gas temperature, 350 °C; dry gas flow, 8 L/min; dry gas temperature, 200 °C; nebuliser pressure, 17 psi; capillary voltage, 3500 V; and nozzle voltage, 1000 V. The untargeted full-scan data were analysed using Agilent Mass Hunter Qualitative Analysis 10.0 and Agilent Mass Hunter Quantitative Analysis (Q-ToF) 10.1. Lipids were identified based on retention time and the isotopic pattern of the mass spectrum, and validated using an authentic in-house MS/MS library, as previously described^37^.

## Data processing and statistical analysis

The metabolite dataset was normalised to the area of internal standards and total protein content from the BCA assay. Multivariate statistical analysis of the quantitative results for each metabolite and lipid was performed using MetaboAnalyst 5.0. All data were pretreated using Pareto scaling and sum normalisation, followed by principal component analysis (PCA) and partial least square-discriminant analysis (PLS-DA). For biomarker selection, variable importance for projection (VIP) values and an S-plot from orthogonal partial least square discriminant analysis (OPLS-DA), Student’s *t*-test, and fold change from a volcano plot (univariate statistical analysis) were used. All supervised statistical analyses (PLS-DA and OPLS-DA) were validated using permutation tests (*p* < 0.05). Bar graphs and other plots were obtained using OriginPro2022 and GraphPad Prism 8. The quantification data of all identified metabolites were employed for pathway mapping using VANTED software (version 2.8.3) based on KEGG pathway.

## Results

### KDM5-C70 induces astrocyte differentiation in NSCs

**Fig. 1:**
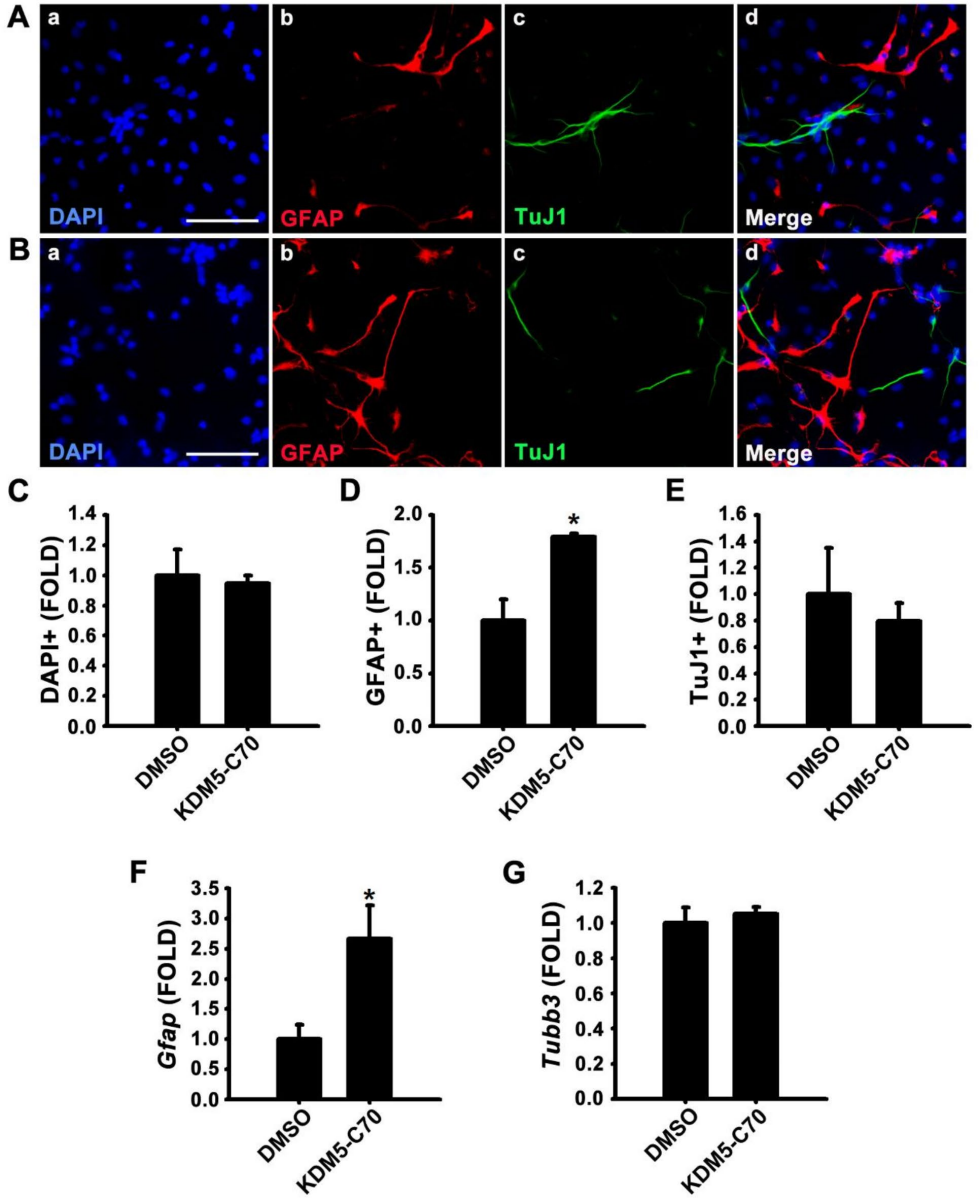
KDM5-C70 increases GFAP expression in rat neural stem cells (NSCs) in the absence of growth factors. (A–E) NSCs treated with 0.1% DMSO or 25 µM KDM5-C70 in the absence of EGF and FGF2 for 4 days. (A, B) Immunofluorescence images of NSCs treated with (A) 0.1% DMSO or (B) 25 µM KDM5-C70 showing (a) nuclei stained with DAPI (blue), (b) astrocytes immuno-stained with GFAP (red), (c) neurons immuno-stained with TuJ1 (green), and (d) merged images. Scale bar = 50 μm. (C-E) Quantification of (C) nuclei, (D) GFAP-positive cells, and (E) TuJ1-positive cells. (F, G) Real-time RT-PCR was performed to quantify the mRNA levels of *Gfap* and *Tubb3*. Total RNA was extracted from NSCs treated with 0.1% DMSO or 25 µM KDM5-C70 in the absence of EGF and FGF2 for 3 days. *Gapdh* was used as an internal control. Data are presented as the mean ± SEM (*n* = 3). * *p* < 0.05, ** *p* < 0.01 (Student’s *t*-test).

NSCs cultured from the cortex of E14 rats proliferate in the presence of EGF and FGF2 and differentiate into neurons, astrocytes, and oligodendrocytes in the absence of these growth factors^13^. To investigate the role of KDM5-C70 in cell fate determination during differentiation, as previously reported^14^, we treated NSCs with either 0.1% DMSO as a control or 25 µM KDM5-C70 without growth factors and performed an ICC (Fig. 1A–E). The astrocytic marker protein GFAP was detected using an anti-GFAP antibody, whereas the neuronal marker protein TUBB3 was detected using a TuJ1 antibody. Total cell numbers were determined with DAPI nuclear staining. KDM5-C70 treatment significantly increased the number of GFAP-positive astrocytes, compared with the control (Fig. 1A–D). However, neuronal differentiation was unaffected by KDM5-C70 treatment (Fig. 1E). Real-time RT-PCR showed that KDM5-C70 treatment increased *Gfap* mRNA expression (Fig. 1F) without affecting *Tubb3* transcript levels (Fig. 1G). These results suggest that KDM5-C70 treatment promotes astrocytogenesis in NSCs.

### Lipidomic perturbation elucidates downregulated PE biosynthesis during astrocytogenesis

Investigation of the effects of KDM5-C70 on astrocytogenesis revealed significant alterations in metabolic pathways and the expression of endogenous metabolites. The differences between the KDM5-C70-treated and control groups were identified through both multivariate and univariate statistical analyses using a GC-MS-based untargeted metabolomic approach. Metabolite profiling identified 42 metabolites associated with key bioenergetic pathways (Table S1), including glycolysis-related metabolites (glucose, sucrose, fructose, 3-phosphoglycerate, and pyruvate), TCA cycle-related metabolites (malate, succinate, and citrate), and amino acids biosynthesized from these pathways (alanine, lysine, tyrosine, cysteine, valine, leucine, serine, glycine, threonine, isoleucine, aspartate, glutamine, and pyroglutamate). Additionally, metabolites linked to the urea cycle (creatinine and putrescine), lipid biosynthesis (palmitic acid, stearic acid, GPA, glycerol, and O-phosphoethanolamine), and phosphate metabolism (UMP, R5P, I1P, and myo-inositol) were detected.

**Fig. 2:**
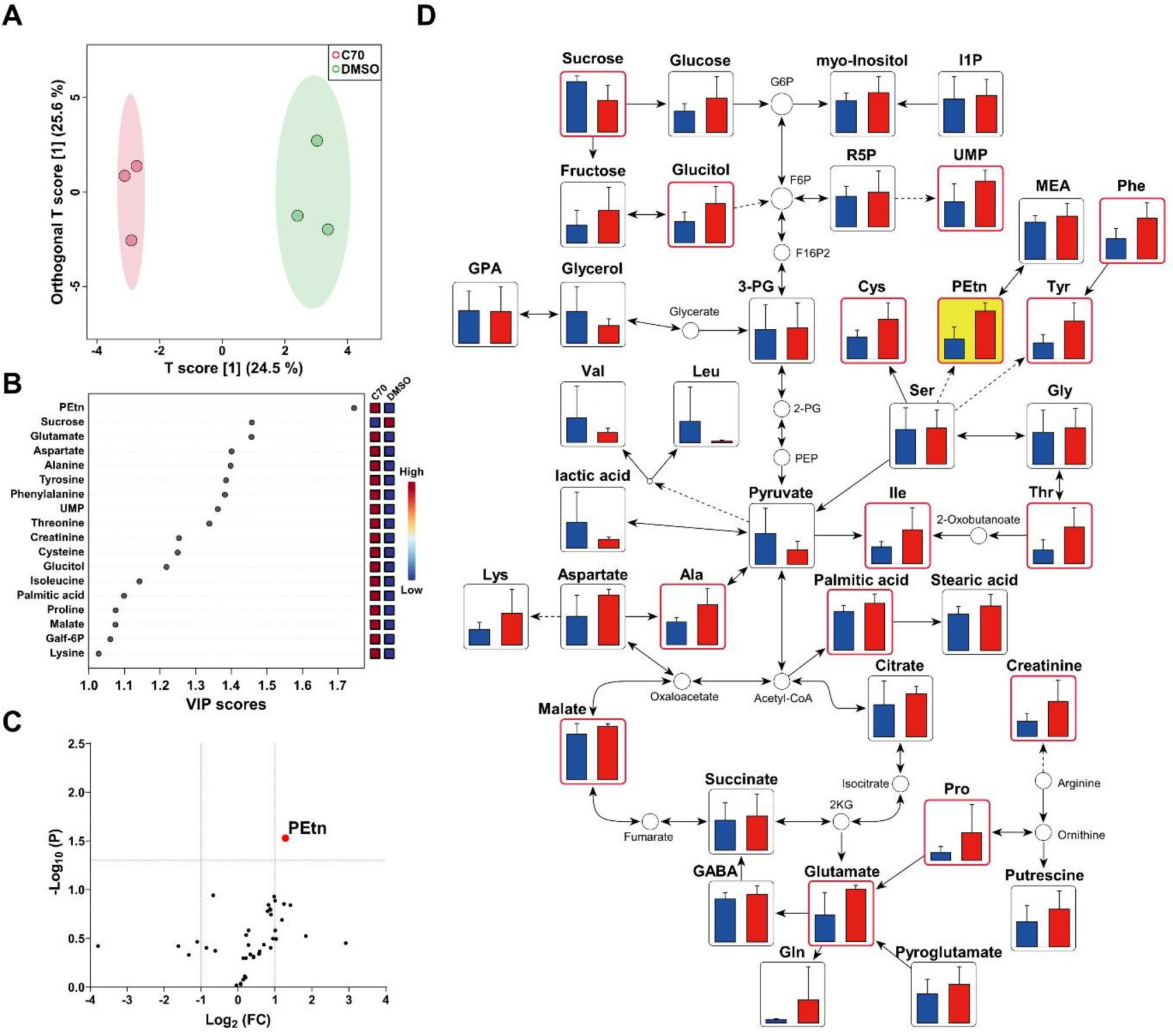
Determination of KDM5-C70 treatment-related metabolites and enriched metabolic networks of endogenous metabolites. (A) OPLS-DA score plot indicating the clustering of the control and KDM5-C70 groups. (B) Metabolites with VIP scores > 1.0. (C) Volcano plot of all identified metabolites. (D) Metabolic network constituted with KDM5-C70 associated metabolic pathways. Metabolites with a VIP score greater than 1.0 are highlighted with a red border. In the bar graph, the control group is represented in blue, whereas the KDM5-C70 group is represented in red. Biomarkers are emphasized with a yellow background.

To identify metabolites with significantly different levels between the sample groups (control, *n* = 3; KDM5-C70, *n* = 3), multivariate and univariate statistical analyses were performed. The OPLS-DA model revealed 18 metabolites with variable importance for projection (VIP) scores exceeding 1.00, indicating distinct separation between groups (Fig. 2A and B). Volcano plot analysis further confirmed that phosphoethanolamine (PEtn) was the most significantly altered metabolite among the groups in the metabolic networks within stem cells (Fig. 2C and D). PEtn accumulated notably during the astrocyte differentiation of NSCs. PEtn, a precursor to PE, is converted into CDP-ethanolamine by the enzyme ethanolamine-phosphate cytidylyltransferase (EPCT), which is encoded by the Pcyt2 gene^38^ and plays a key role in phospholipid metabolism during astrocyte differentiation. This implies that KDM5-C70 affects phospholipid biosynthesis, leading to alterations in cell morphology and composition.

**Fig. 3:**
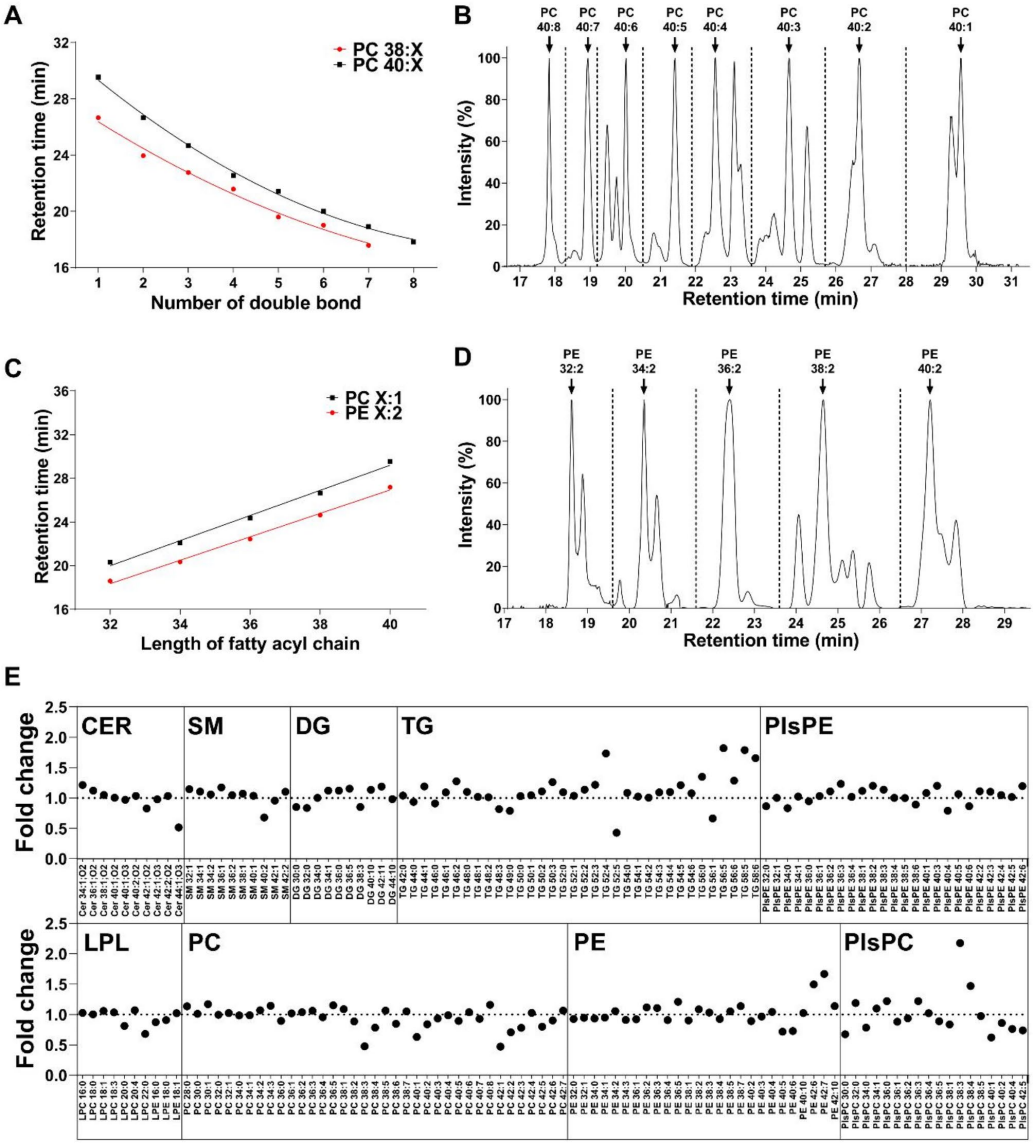
Lipid identification based on the distribution of retention time relative to the number of carbons and unsaturated bonds in the fatty acyl chain. (A) Quadratic regression plot of PC 38 (X ranges from 1 to 7) and PC 40 (X ranges from 1 to 8). (B) PC 40 chromatogram. (C) Linear regression plot of PC X:1 (X ranged from 32 to 40) and PE X:2 (X ranged from 32 to 40). (D) PE X:2 chromatogram. (E) Fold change plot of all identified lipids.

Lipidomic profiling using an in-house library resulted in the detection of 180 lipids in NSC. Under optimised LC-MS conditions, all lipid species exhibited a consistent retention time interval pattern, correlating with the changes in the number of unsaturated bonds (Fig. 3A and B) or the length of the fatty acyl chains (Fig. 3C and D) of the lipids. This pattern, combined with spectral comparison with our in-house library, enabled accurate lipid identification. QC samples were used to identify each lipid in both groups, and 180 lipids were detected, including 10 ceramides (Cer), 14 sphingomyelins (SM), 12 diacylglycerols (DG), 34 triacylglycerols (TG), 28 PE, 40 phosphatidylcholines (PC), 25 plasmenyl-phosphatidylethanolamines (PlsPE), 18 plasmenyl-phosphatidylcholines (PlsPC), and 10 lysophospholipids (LPL), whose fold changes were represented in Fig. 3E.

**Fig. 4:**
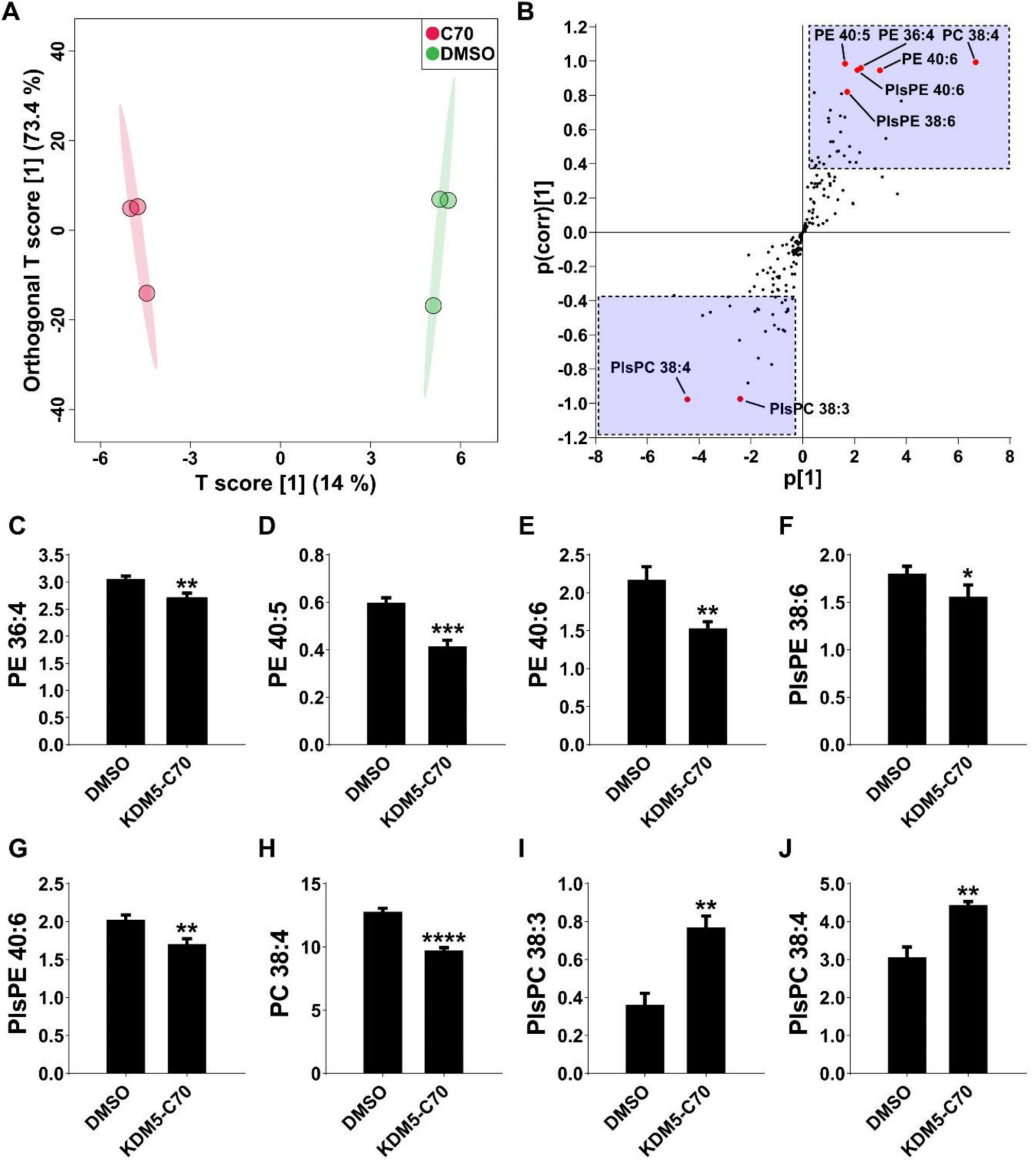
Treatment with KDM5-C70 induced significant alterations in various lipids,** with pronounced changes in phospholipids.** (A) OPLS-DA score plot demonstrating separation between the control and KDM5-C70 groups. (B) S-plot derived from the OPLS-DA model indicating the highly correlated putative lipids. Lipids with a VIP score greater than 1.0 are in the blue region, whereas biomarkers are indexed by a red symbol. Table S12 shows the statistical analysis results of lipids in the blue region. (C–J) Normalized intensity of lipid biomarkers: (C) PE 36:4, (D) PE 40:5, (E) PE 40:6, (F) PlsPE 38:6, (G) PlsPE 40:6, (H) PC 38:4, (I) PlsPC 38:3, and (J) PlsPC 38:4. * *p* < 0.05, ** *p* < 0.01, *** *p* < 0.001, **** *p* < 0.0001 (Student’s *t*-test).

The biosynthesis of phospholipids with unsaturated fatty acyl chains, including PE and PC, was downregulated during KDM5-C70-induced astrocytogenesis. Using multivariate and univariate statistical analyses of lipid profiles, we identified the lipid species altered by KDM5-C70 treatment. A clear clustering between the control and KDM5-C70 groups was observed in the OPLS-DA score plot (Fig. 4A), with 59 lipids distinguishing the two groups based on their VIP scores (Table S12). The OPLS-DA S-plot and t-test revealed significant changes in eight lipids in KDM5-C70-differentiated stem cells (Fig. 4B and Table S12). Among them, the PE species (PE 36:4, PE 40:5, and PE 40:6) and ether-linked PE (PlsPE 38:6 and PlsPE 40:6) decreased in the KDM5-C70 group (Fig. 4C–G). Similarly, PC 38:4 levels decreased in the KDM5-C70 group (Fig. 4H), whereas two PlsPC species accumulated significantly (Fig. 4I–J).

### KDM5-C70 regulates the expression of genes responsible for PE biosynthesis

**Fig. 5:**
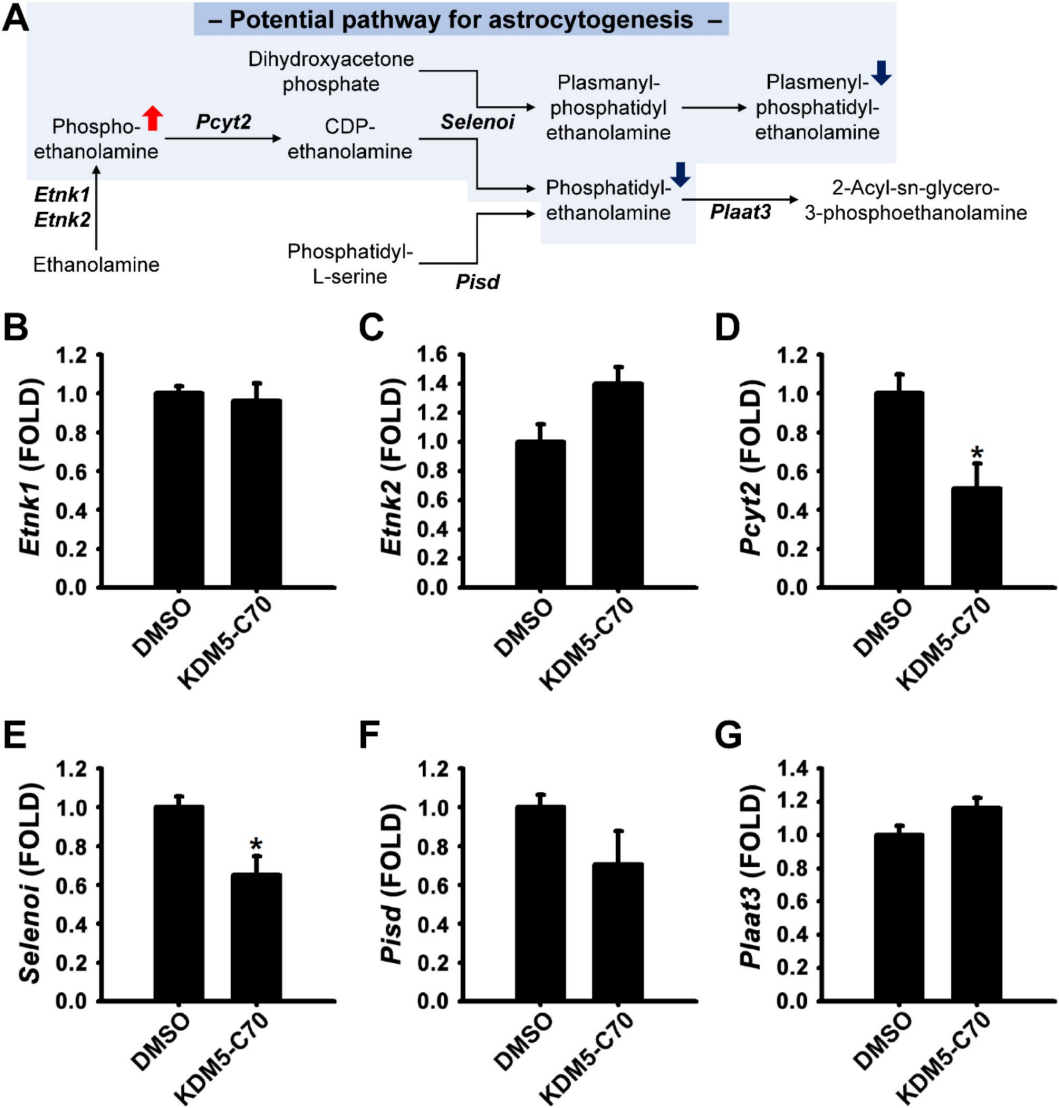
mRNA expression levels of genes involved in PE biosynthesis. (A) PE biosynthesis metabolism and its associated regulators. (B–G) *Etnk1*, *Etnk2*, *Pcyt2*, *Selenoi*, *Pisd*, and *Plaat3* mRNA expression levels were determined using real-time RT-PCR. Total RNA was extracted from NSCs treated with 0.1% DMSO or 25 µM KDM5-C70 in the absence of EGF and FGF2 for 3 days. *Gapdh* was used as an internal control. Data are presented as the mean ± SEM (*n* = 3). * *p* < 0.05, ** *p* < 0.01 (Student’s *t*-test).

We explored the mechanisms by which KDM5-C70 modulated PEtn and PE perturbations by examining gene expression within the PE biosynthetic pathway (Fig. 5A). The genes *Etnk1*, *Etnk2*, *Pcyt2*, *Selenoi*, *Pisd*, and *Plaat3*, which encode enzymes critical for PE biosynthesis, were analysed. Gene expression levels were assessed in differentiated cells treated with either 0.1% DMSO or 25 µM KDM5-C70 for 3 days. KDM5-C70 treatment significantly reduced the mRNA levels of *Pcyt2* and *Selenoi* but did not markedly affect the transcript levels of *Etnk1*, *Etnk2*, *Pisd*, and *Plaat3* (Fig. 5B–G). These findings suggest that KDM5-C70 inhibits the expression of specific biosynthetic enzymes, leading to increased PEtn and decreased PE and PlsPE levels.

## Discussion

In our study, we utilised metabolomics and lipidomics to uncover the mechanisms pivotal in NSC differentiation induced by the KDM inhibitor KDM5-C70. As previously reported, KDM5-C70 promotes astrocytogenesis in NSCs^14^. Unlike earlier studies that focused primarily on the biological and cellular effects, especially gene expression, following KDM5-C70 treatment^39,40^, our study aimed to elucidate the roles of metabolites and lipids. We hypothesised that energy metabolism, which is essential for cell differentiation^41^, and structural lipids, particularly phospholipids, are instrumental in mediating changes in cell structure. This approach aimed to expand our understanding beyond genetic alterations by investigating the contribution of metabolic and lipidomic dynamics to cellular differentiation.

Our integrative metabolomic and lipidomic analyses revealed that KDM5-C70 treatment downregulated PE biosynthesis during astrocyte differentiation. Using GC-MS and LC-MS, we identified significant differences between metabolites and lipids within the metabolic network, particularly in the differentiation of PEtn and its products, PE lipids. The accumulation of PEtn appeared to result from the decreased expression of *Pcyt2*, which converts PEtn to CDP-PEtn, and *selenoi*, which catalyses the conversion of CDP-PEtn to PE (Fig. 5D–E). The lack of significant upregulation in the expression of the *plaat3* gene, involved in PE catabolism, suggests that reduced expression of *Pcyt2* and *Selenoi* directly causes decreased PE lipid levels during astrocyte differentiation (Fig. 5G). The decrease in *Pcyt2* expression and subsequent accumulation of PEtn are known to confer protection against glutamine deprivation in cancer cells, indicating the critical role of *Pcyt2* as a rate-limiting step in PE biosynthesis and its impact on the overall decrease in PE lipid species^42^. The significance of PEtn and its related genes in brain and neural cell functions has been a subject of research. Studies, including those on the *Drosophila* mutant easily shocked (eas), which exhibits seizures due to disrupted phospholipid metabolism affecting membrane composition, underline the importance of PEtn in nervous system functionality^43^. Additionally, variations in the expression of Etnppl, predominantly located in the brain and liver, have been associated with various neurological and hepatic disorders, emphasising its role in lipid synthesis and its potential for therapeutic intervention in conditions such as schizophrenia, bipolar disorder, and certain cancers^44–46^.

Montaner et al., reported that the administration of exogenous PE-enriched liposomes promotes astrocyte differentiation and maturation^47^. These findings are different from our results that show that endogenous PE levels are reduced during astrocyte differentiation. The observed discrepancy may be attributed to differences in cell lines and culture conditions. In Montaner et al’s study, neurospheres were isolated from embryonic brains of E13 timed-pregnant mice^47^. Then cells were plated without adding growth factors, and PE was supplemented simultaneously, which enhanced astrocyte differentiation from post-mitotic cells^47^. In contrast, in our study, we used NSCs isolated from the embryonic brains of E14 rats. Neurospheres were expanded for six days in the presence of growth factors, and cells were plated. After a 24-hour period, NSCs were treated with KDM5-C70 in the absence of growth factors to induce differentiation. These differences in cell origin, culture conditions, and timing of treatments likely contribute to the differing outcomes observed.

Another reason that Montaner’s study^47^ and ours are different might be from the composition of the fatty acids of PE. In our study, the PE species we observed to decrease during astrocytogenesis have a higher degree of unsaturation, with 4 to 6 double bonds (PE 36:4, PE 40:5, PE 40:6). This is different from the exogenous PE used in Montaner et al.'s study, which had a different balance of saturated and unsaturated fatty acids (approximately 50% saturated and 50% mono- and di-unsaturated fatty acids)^47^. Given these differences, the contrasting effects of PE on astrocyte differentiation in the two studies could reflect not only the experimental conditions but also the specific fatty acid composition of the PE species involved. This suggests that not only the presence of PE lipid species but also the specific composition of fatty acids within the PE plays an important role in the differentiation of NSCs into astrocytes.

There are reports that during cellular differentiation, endogenous PE levels increase in other cell types, such as cardiomyocytes^48^ and osteoclasts^49^ These and our results suggest that alterations in PE levels are a recognized feature of cellular differentiation processes. It is possible that PE plays a critical role in the early stages of astrocyte differentiation, and the observed decrease in PE levels may reflect reduced demand for PE as astrocyte differentiation progress. Given that the role of PE in various stages of astrocyte differentiation and maturation is not yet fully understood, further studies, including time-course experiments and more specific cell culture models, will be necessary.

There are just a few research concerning the regulation of genes or enzymes involved in PE biosynthesis and subsequent changes in stem cell differentiation. Bogdanovic et al. have suggested, based on gene silencing studies, that IDH1—which facilitates the conversion of isocitrate to alpha-ketoglutarate—indirectly influences phospholipid metabolism^50^. Specifically, their research demonstrated that siRNA-mediated knockdown of IDH1 impairs PE synthesis, thereby affecting astrocyte differentiation. Although our data suggest that downregulation of PE biosynthesis correlates with astrocyte differentiation, it is not yet clear whether the reduction in PE levels causes or results from astrocytogenesis. Future studies using inhibitors of key enzymes in the PE biosynthesis pathway, such as Pcyt2, are needed to determine if reduced PE levels induce astrocytogenesis or are simply a result of the differentiation process. Meclizine is an inhibitor of Pcyt2 and has been used to impair PE biosynthesis^51^. Small molecules, namely PCiB-2, -3, and − 4, have been discovered as inhibitors of PE methyltransferase activity^52^. These inhibitors could be useful in our future studies to investigate the functional role of PE during astrocytogenesis.

A noteworthy finding of our study was the predominant reduction in PE lipids containing polyunsaturated fatty acyl chains. This suggests that lipids with a high degree of unsaturation in fatty acyl chains play a crucial role in stem cell differentiation, particularly in astrocyte differentiation. Numerous studies have reported that unsaturated fatty acids are notably involved in the differentiation of stem cells into various cell types. Particularly, the administration of omega-3 fatty acids, such as eicosapentaenoic acid (EPA) and docosahexaenoic acid (DHA) has been shown to induce differentiation of NSCs into all potential cell types, including neurons, astrocytes, and oligodendrocytes. Patient-derived NSC treated with EPA and DHA induced the differentiation of rat NSCs into astrocytes^53^. Changes in such polyunsaturated fatty acids (PUFAs) have also played a significant role in the differentiation of neural stem cells into neurons, whereas omega-6 arachidonic acid does not demonstrate the same pattern^54^. Unsaturated fats are being elucidated not only for their role in stem cell differentiation but also for their involvement in cellular structure and organelle function. Additionally, the unsaturation of lipid acyl chains is reported to be crucial in cellular formation. The unsaturation process shapes the nuclear envelope and significantly influences the architecture of the nuclear pore complex, which serves as a conduit for interactions between the nucleus and the cytoplasm^55,56^. This demonstrates that, in addition to the biosynthesis of the PE lipid, the unsaturated fatty acids composing the fatty acyl chains of discovered lipid biomarkers could also play a crucial role in the differentiation of stem cells. Integrative interpretation of our findings on PEtn with those of previous studies on PUFAs could suggest further studies for exploring the relationship between a quantitative reduction in unsaturated fat and NSC differentiation.

Research on various astrocyte-differentiating agents elucidates the mechanisms underlying induced differentiation. Our study revealed changes in mechanisms caused by a novel KDM5 inhibitor KDM5-C70, highlighting the potential role of phospholipids, particularly PE, in differentiation processes. However, it remains to be determined whether the mechanisms by other agents are similar. Specifically, examination of metabolites after treatment with other KDM5 inhibitors, such as CPI-455, which has recently been shown to enhance astrocytogenesis in NSCs, would be interesting to pursue^15^ Therefore, it is crucial to observe the changes in biochemical metabolites and lipidomics, to understand their roles in differentiation.

## Conclusion

Our comprehensive metabolomic and lipidomic analyses elucidated the pivotal role of KDM5-C70 in the differentiation of NSCs into astrocytes, focusing on the significant downregulation of PE biosynthesis. This effect is mediated by the accumulation of PEtn and subsequent reduction in the expression of genes essential for its conversion to PE, underscoring the importance of phospholipid metabolism in astrocytogenesis. Specifically, we found that PEtn accumulation, caused by the decreased expression of *Pcyt2* and *Selenoi*, led to a notable decrease in PE lipids, particularly those containing polyunsaturated fatty acyl chains, which potentially play a crucial role in cell differentiation. These findings suggest that downregulation of PE biosynthesis and its impact on lipid composition may be associated with KDM5-C70-mediated astrocyte differentiation. Our study not only provides new insights into the molecular mechanism of astrocytogenesis but also highlights the potential of targeting phospholipid metabolism pathways as therapeutic strategies for diseases involving dysregulated astrocyte function.

## Electronic supplementary material

Below is the link to the electronic supplementary material.

Supplementary Material 1

## Data Availability

The datasets used and/or analysed during the current study available from the corresponding author on reasonable request.
