# Supplementary material for: Histone demethylase inhibitor KDM5-C70 regulates metabolomic and lipidomic programming during an astrocyte differentiation of rat neural stem cell
Source: Sci Rep. 2025 Feb 13;15:5409. doi: 10.1038/s41598-025-88636-7 (PMC11825845; doi:10.1038/s41598-025-88636-7)
Supplement: Supplementary file 1 — Supplementary Material 1 [file 41598_2025_88636_MOESM1_ESM.docx]

Histone demethylase inhibitor KDM5-C70 regulates metabolomic and lipidomic programming during an astrocyte differentiation of rat neural stem cell

Minki Shim, Thin Thin San, Bohyun Shin, Hyojeong Lee, Sang Beom Han, Dong-Kyu Lee, Hyun-Jung Kim

**Table S1. List of identified metabolites**

| No. | Metabolite | Retention index | Retention time (min) | TMS | Quantification ion (m/z) |
| --- | --- | --- | --- | --- | --- |
| 1 | Pyruvic acid | 1066 | 8.01 | 1 | 174 |
| 2 | Lactic acid | 1073 | 8.29 | 2 | 73 |
| 3 | Alanine | 1104 | 9.57 | 2 | 116 |
| 4 | Leucine | 1151 | 11.50 | 1 | 86 |
| 5 | Monomethylphosphate | 1173 | 12.38 | 2 | 241 |
| 6 | Valine | 1214 | 14.17 | 2 | 144 |
| 7 | Aminoethanol | 1263 | 16.52 | 3 | 174 |
| 8 | Glycerol | 1276 | 17.12 | 3 | 73 |
| 9 | Isoleucine | 1291 | 17.86 | 2 | 158 |
| 10 | Proline | 1293 | 17.93 | 2 | 142 |
| 11 | Glycine | 1302 | 18.37 | 3 | 176 |
| 12 | Succinic acid | 1315 | 18.98 | 2 | 147 |
| 13 | Serine | 1361 | 21.09 | 3 | 73 |
| 14 | Threonine | 1385 | 22.19 | 3 | 73 |
| 15 | Malic acid | 1490 | 26.78 | 3 | 73 |
| 16 | Pyroglutamic acid | 1514 | 27.78 | 2 | 156 |
| 17 | Aspartic acid | 1521 | 28.06 | 3 | 73 |
| 18 | 4-Aminobutyric acid | 1525 | 28.24 | 3 | 174 |
| 19 | Creatinine | 1544 | 28.97 | 3 | 115 |
| 20 | Cysteine | 1552 | 29.33 | 3 | 220 |
| 21 | Phenlyalanine | 1617 | 31.95 | 2 | 73 |
| 22 | Glutamic acid | 1621 | 32.08 | 3 | 73 |
| 23 | Putrescine | 1728 | 36.22 | 4 | 174 |
| 24 | alpha-Glycerophosphate | 1765 | 37.50 | 4 | 73 |
| 25 | L-Glutamine | 1773 | 37.78 | 3 | 73 |
| 26 | O-Phosphorylethanolamine | 1778 | 37.98 | 4 | 73 |
| 27 | 3-Phosphoglyceric acid | 1807 | 38.96 | 4 | 73 |
| 28 | Citric acid | 1819 | 39.32 | 4 | 73 |
| 29 | D-Fructose | 1870 | 40.87 | 1 | 73 |
| 30 | Glucose | 1909 | 42.05 | 5 | 73 |
| 31 | L-Lysine | 1917 | 42.29 | 4 | 73 |
| 32 | Glucitol | 1929 | 42.62 | 6 | 319 |
| 33 | L-Tyrosine | 1932 | 42.71 | 3 | 218 |
| 34 | Palmitic acid | 2049 | 46.00 | 1 | 73 |
| 35 | Inositol | 2082 | 46.94 | 6 | 73 |
| 36 | D-Ribose-5-Phosphate | 2106 | 47.58 | 5 | 73 |
| 37 | Steric acid | 2244 | 50.23 | 1 | 73 |
| 38 | D-Galactofuranose-6-P | 2298 | 50.12 | 6 | 73 |
| 39 | L-myo-Inositol-2-Phosphate | 2399 | 52.54 | 7 | 73 |
| 40 | Inosine | 2564 | 54.49 | 4 | 73 |
| 41 | Sucrose | 2623 | 55.14 | 8 | 73 |
| 42 | 5-Uridine P | 3041 | 59.15 | 5 | 73 |

**Table S2. Statistical analysis results of polar metabolites**

| No. | Metabolites | VIP value | *p*-value | Fold change |
| --- | --- | --- | --- | --- |
| 1 | O-Phosphorylethanolamine | 1.74 | 0.03 | 2.44 |
| 2 | Sucrose | 1.46 | 0.11 | 0.63 |
| 3 | Glutamic acid | 1.46 | 0.12 | 1.97 |
| 4 | Aspartic acid | 1.40 | 0.17 | 1.74 |
| 5 | Alanine | 1.40 | 0.14 | 1.77 |
| 6 | L-Tyrosine | 1.39 | 0.14 | 2.37 |
| 7 | Phenlyalanine | 1.38 | 0.13 | 2.01 |
| 8 | 5-Uridine P | 1.36 | 0.16 | 1.84 |
| 9 | Threonine | 1.34 | 0.14 | 2.67 |
| 10 | Creatinine | 1.25 | 0.20 | 2.28 |
| 11 | Cysteine | 1.25 | 0.16 | 1.82 |
| 12 | Glucitol | 1.22 | 0.18 | 1.86 |
| 13 | Isoleucine | 1.14 | 0.26 | 2.01 |
| 14 | Palmitic acid | 1.10 | 0.26 | 1.22 |
| 15 | Proline | 1.08 | 0.30 | 3.57 |
| 16 | Malic acid | 1.07 | 0.29 | 1.17 |
| 17 | D-Galactofuranose-6-P | 1.06 | 0.32 | 1.93 |
| 18 | L-Lysine | 1.03 | 0.32 | 2.05 |
| 19 | Pyruvic acid | 0.94 | 0.34 | 0.47 |
| 20 | L-Glutamine | 0.93 | 0.35 | 7.56 |
| 21 | Leucine | 0.92 | 0.38 | 0.07 |
| 22 | Steric acid | 0.90 | 0.37 | 1.22 |
| 23 | Lactic acid | 0.90 | 0.38 | 0.33 |
| 24 | Glucose | 0.88 | 0.37 | 1.63 |
| 25 | Inosine | 0.88 | 0.46 | 1.48 |
| 26 | Glycerol | 0.84 | 0.39 | 0.55 |
| 27 | D-Fructose | 0.82 | 0.40 | 1.84 |
| 28 | Aminoethanol | 0.82 | 0.50 | 1.16 |
| 29 | Valine | 0.76 | 0.47 | 0.40 |
| 30 | Citric acid | 0.75 | 0.49 | 1.34 |
| 31 | Inositol | 0.73 | 0.46 | 1.26 |
| 32 | Putrescine | 0.72 | 0.43 | 1.50 |
| 33 | Monomethylphosphate | 0.72 | 0.42 | 0.65 |
| 34 | Pyroglutamic acid | 0.63 | 0.49 | 1.34 |
| 35 | 4-Aminobutyric acid | 0.62 | 0.50 | 1.10 |
| 36 | D-Ribose-5-Phosphate | 0.36 | 0.77 | 1.14 |
| 37 | Succinic acid | 0.36 | 0.81 | 1.14 |
| 38 | L-myo-Inositol-2-Phosphate | 0.34 | 0.84 | 1.10 |
| 39 | Glycine | 0.16 | 0.79 | 1.15 |
| 40 | 3-Phosphoglyceric acid | 0.09 | 0.94 | 1.06 |
| 41 | Serine | 0.07 | 0.92 | 1.05 |
| 42 | alpha-Glycerophosphate | 0.02 | 0.96 | 0.97 |

**Table S3. List of identified lipids in diacylglycerols**

| No. | Identity | Retention time (min) | Adduct ion | Quantification ion (m/z) |
| --- | --- | --- | --- | --- |
| 1 | DG 30:0 | 23.36 | [M+NH_4_]^+^ | 558.509 |
| 2 | DG 32:0 | 26.00 | [M+NH_4_]^+^ | 586.541 |
| 3 | DG 34:0 | 28.30 | [M+NH_4_]^+^ | 614.572 |
| 4 | DG 34:1 | 25.80 | [M+NH_4_]^+^ | 612.556 |
| 5 | DG 34:5 | 24.74 | [M+NH_4_]^+^ | 604.494 |
| 6 | DG 36:0 | 30.57 | [M+NH_4_]^+^ | 642.603 |
| 7 | DG 36:5 | 27.55 | [M+NH_4_]^+^ | 632.525 |
| 8 | DG 38:3 | 27.42 | [M+NH_4_]^+^ | 664.588 |
| 9 | DG 40:10 | 18.28 | [M+NH_4_]^+^ | 679.509 |
| 10 | DG 42:11 | 18.30 | [M+NH_4_]^+^ | 704.525 |
| 11 | DG 44:9 | 20.50 | [M+NH_4_]^+^ | 736.588 |
| 12 | DG 44:10 | 22.26 | [M+NH_4_]^+^ | 734.572 |

**Table S4. List of identified lipids in triacylglycerols**

| No. | Identity | Retention time (min) | Adduct ion | Quantification ion (m/z) |
| --- | --- | --- | --- | --- |
| 1 | TG 42:0 | 32.73 | [M+NH_4_]^+^ | 740.676 |
| 2 | TG 44:0 | 33.53 | [M+NH_4_]^+^ | 768.708 |
| 3 | TG 44:1 | 32.76 | [M+NH_4_]^+^ | 766.692 |
| 4 | TG 46:0 | 34.23 | [M+NH_4_]^+^ | 796.739 |
| 5 | TG 46:1 | 33.53 | [M+NH_4_]^+^ | 794.723 |
| 6 | TG 46:2 | 32.80 | [M+NH_4_]^+^ | 792.708 |
| 7 | TG 48:0 | 34.93 | [M+NH_4_]^+^ | 824.770 |
| 8 | TG 48:1 | 34.10 | [M+NH_4_]^+^ | 822.755 |
| 9 | TG 48:2 | 33.41 | [M+NH_4_]^+^ | 820.739 |
| 10 | TG 48:3 | 32.80 | [M+NH_4_]^+^ | 818.723 |
| 11 | TG 49:0 | 35.10 | [M+Na]^+^ | 843.741 |
| 12 | TG 50:0 | 35.67 | [M+NH_4_]^+^ | 852.802 |
| 13 | TG 50:1 | 34.77 | [M+NH_4_]^+^ | 850.786 |
| 14 | TG 50:2 | 33.98 | [M+NH_4_]^+^ | 848.770 |
| 15 | TG 50:3 | 33.45 | [M+NH_4_]^+^ | 846.755 |
| 16 | TG 52:0 | 36.49 | [M+NH_4_]^+^ | 880.833 |
| 17 | TG 52:1 | 35.48 | [M+NH_4_]^+^ | 878.817 |
| 18 | TG 52:2 | 34.62 | [M+NH_4_]^+^ | 876.802 |
| 19 | TG 52:3 | 33.95 | [M+NH_4_]^+^ | 874.786 |
| 20 | TG 52:4 | 34.62 | [M+Na]^+^ | 872.770 |
| 21 | TG 52:5 | 32.63 | [M+NH_4_]^+^ | 870.755 |
| 22 | TG 54:0 | 37.39 | [M+NH_4_]^+^ | 908.864 |
| 23 | TG 54:1 | 36.25 | [M+NH_4_]^+^ | 906.848 |
| 24 | TG 54:2 | 35.32 | [M+NH_4_]^+^ | 904.833 |
| 25 | TG 54:3 | 34.50 | [M+NH_4_]^+^ | 902.817 |
| 26 | TG 54:4 | 33.85 | [M+NH_4_]^+^ | 900.802 |
| 27 | TG 54:5 | 33.18 | [M+NH_4_]^+^ | 898.786 |
| 28 | TG 54:6 | 32.44 | [M+NH_4_]^+^ | 896.770 |
| 29 | TG 56:0 | 38.37 | [M+NH_4_]^+^ | 936.895 |
| 30 | TG 56:1 | 37.09 | [M+NH_4_]^+^ | 934.880 |
| 31 | TG 56:5 | 33.73 | [M+NH_4_]^+^ | 926.817 |
| 32 | TG 56:6 | 33.00 | [M+NH_4_]^+^ | 924.802 |
| 33 | TG 58:5 | 34.32 | [M+NH_4_]^+^ | 954.848 |
| 34 | TG 58:6 | 34.08 | [M+NH_4_]^+^ | 952.833 |

**Table S5. List of identified lipids in ceramides**

| No. | Identity | Retention time (min) | Adduct ion | Quantification ion (m/z) |
| --- | --- | --- | --- | --- |
| 1 | Cer 34:1;O2 | 22.24 | [M+H]^+^ | 538.519 |
| 2 | Cer 36:1;O2 | 24.74 | [M+H]^+^ | 566.551 |
| 3 | Cer 38:1;O2 | 27.53 | [M+H]^+^ | 594.582 |
| 4 | Cer 40:1;O2 | 29.44 | [M+H]^+^ | 622.613 |
| 5 | Cer 40:1;O3 | 21.18 | [M+H]^+^ | 638.608 |
| 6 | Cer 40:2;O2 | 26.92 | [M+H]^+^ | 620.598 |
| 7 | Cer 42:1;O2 | 31.69 | [M+H]^+^ | 650.645 |
| 8 | Cer 42:1;O3 | 23.40 | [M+H]^+^ | 666.640 |
| 9 | Cer 42:2;O2 | 29.71 | [M+H]^+^ | 648.629 |
| 10 | Cer 44:1;O3 | 25.79 | [M+H]^+^ | 694.671 |

**Table S6. List of identified lipids in sphingomyelins**

| No. | Identity | Retention time (min) | Adduct ion | Quantification ion (m/z) |
| --- | --- | --- | --- | --- |
| 1 | SM 32:1 | 18.58 | [M+H]^+^ | 675.544 |
| 2 | SM 34:0 | 21.37 | [M+H]^+^ | 705.591 |
| 3 | SM 34:1 | 20.51 | [M+H]^+^ | 703.575 |
| 4 | SM 34:2 | 18.63 | [M+H]^+^ | 701.560 |
| 5 | SM 36:0 | 23.84 | [M+H]^+^ | 733.622 |
| 6 | SM 36:1 | 22.81 | [M+H]^+^ | 731.607 |
| 7 | SM 36:2 | 20.52 | [M+H]^+^ | 729.591 |
| 8 | SM 38:0 | 26.58 | [M+H]^+^ | 761.654 |
| 9 | SM 38:1 | 25.39 | [M+H]^+^ | 759.638 |
| 10 | SM 40:0 | 29.39 | [M+H]^+^ | 789.685 |
| 11 | SM 40:1 | 28.20 | [M+H]^+^ | 787.669 |
| 12 | SM 40:2 | 24.96 | [M+H]^+^ | 785.654 |
| 13 | SM 42:1 | 30.54 | [M+H]^+^ | 815.701 |
| 14 | SM 42:2 | 27.63 | [M+H]^+^ | 813.685 |

**Table S7. List of identified lipids in phosphatidylcholines**

| No. | Identity | Retention time (min) | Adduct ion | Quantification ion (m/z) |
| --- | --- | --- | --- | --- |
| 1 | PC 28:0 | 18.28 | [M+H]^+^ | 678.507 |
| 2 | PC 30:0 | 20.04 | [M+H]^+^ | 706.539 |
| 3 | PC 30:1 | 18.30 | [M+H]^+^ | 704.523 |
| 4 | PC 32:0 | 22.26 | [M+H]^+^ | 734.570 |
| 5 | PC 32:1 | 20.18 | [M+H]^+^ | 732.554 |
| 6 | PC 32:2 | 18.56 | [M+H]^+^ | 730.539 |
| 7 | PC 32:3 | 17.85 | [M+H]^+^ | 728.523 |
| 8 | PC 34:0 | 24.59 | [M+H]^+^ | 762.601 |
| 9 | PC 34:1 | 22.16 | [M+H]^+^ | 760.586 |
| 10 | PC 34:2 | 20.27 | [M+H]^+^ | 758.570 |
| 11 | PC 34:3 | 19.22 | [M+H]^+^ | 756.554 |
| 12 | PC 34:4 | 18.11 | [M+H]^+^ | 754.539 |
| 13 | PC 36:0 | 27.28 | [M+H]^+^ | 790.633 |
| 14 | PC 36:1 | 24.46 | [M+H]^+^ | 788.617 |
| 15 | PC 36:2 | 21.84 | [M+H]^+^ | 786.601 |
| 16 | PC 36:3 | 21.30 | [M+H]^+^ | 784.586 |
| 17 | PC 36:4 | 19.77 | [M+H]^+^ | 782.570 |
| 18 | PC 36:5 | 18.13 | [M+H]^+^ | 780.554 |
| 19 | PC 38:1 | 26.85 | [M+H]^+^ | 816.648 |
| 20 | PC 38:2 | 24.11 | [M+H]^+^ | 814.633 |
| 21 | PC 38:3 | 22.87 | [M+H]^+^ | 812.617 |
| 22 | PC 38:4 | 21.70 | [M+H]^+^ | 810.601 |
| 23 | PC 38:5 | 19.65 | [M+H]^+^ | 808.586 |
| 24 | PC 38:6 | 19.08 | [M+H]^+^ | 806.570 |
| 25 | PC 38:7 | 17.65 | [M+H]^+^ | 804.554 |
| 26 | PC 40:1 | 29.69 | [M+H]^+^ | 844.680 |
| 27 | PC 40:2 | 26.85 | [M+H]^+^ | 842.664 |
| 28 | PC 40:3 | 24.81 | [M+H]^+^ | 840.648 |
| 29 | PC 40:4 | 22.71 | [M+H]^+^ | 838.633 |
| 30 | PC 40:5 | 21.52 | [M+H]^+^ | 836.617 |
| 31 | PC 40:6 | 20.10 | [M+H]^+^ | 834.601 |
| 32 | PC 40:7 | 19.03 | [M+H]^+^ | 832.586 |
| 33 | PC 40:8 | 17.88 | [M+H]^+^ | 830.570 |
| 34 | PC 42:1 | 31.33 | [M+H]^+^ | 872.711 |
| 35 | PC 42:2 | 29.27 | [M+H]^+^ | 870.695 |
| 36 | PC 42:3 | 26.67 | [M+H]^+^ | 868.680 |
| 37 | PC 42:4 | 24.61 | [M+H]^+^ | 866.664 |
| 38 | PC 42:5 | 23.79 | [M+H]^+^ | 864.648 |
| 39 | PC 42:6 | 21.40 | [M+H]^+^ | 862.633 |
| 40 | PC 42:7 | 20.30 | [M+H]^+^ | 860.617 |

**Table S8. List of identified lipids in phosphatidylehtanolamine**

| No. | Identity | Retention time (min) | Adduct ion | Quantification ion (m/z) |
| --- | --- | --- | --- | --- |
| 1 | PE 32:0 | 22.77 | [M+H]^+^ | 692.523 |
| 2 | PE 32:1 | 20.52 | [M+H]^+^ | 690.507 |
| 3 | PE 32:2 | 18.72 | [M+H]^+^ | 688.492 |
| 4 | PE 34:0 | 25.30 | [M+H]^+^ | 720.554 |
| 5 | PE 34:1 | 22.66 | [M+H]^+^ | 718.539 |
| 6 | PE 34:2 | 20.47 | [M+H]^+^ | 716.523 |
| 7 | PE 34:3 | 18.95 | [M+H]^+^ | 714.507 |
| 8 | PE 36:1 | 25.10 | [M+H]^+^ | 746.570 |
| 9 | PE 36:2 | 22.56 | [M+H]^+^ | 744.554 |
| 10 | PE 36:3 | 20.72 | [M+H]^+^ | 742.539 |
| 11 | PE 36:4 | 20.22 | [M+H]^+^ | 740.523 |
| 12 | PE 36:5 | 18.50 | [M+H]^+^ | 738.507 |
| 13 | PE 38:1 | 27.68 | [M+H]^+^ | 774.601 |
| 14 | PE 38:2 | 24.79 | [M+H]^+^ | 772.586 |
| 15 | PE 38:3 | 24.21 | [M+H]^+^ | 770.570 |
| 16 | PE 38:4 | 22.29 | [M+H]^+^ | 768.554 |
| 17 | PE 38:5 | 20.13 | [M+H]^+^ | 766.539 |
| 18 | PE 38:7 | 17.96 | [M+H]^+^ | 762.507 |
| 19 | PE 40:2 | 27.85 | [M+H]^+^ | 800.617 |
| 20 | PE 40:3 | 26.03 | [M+H]^+^ | 798.601 |
| 21 | PE 40:4 | 23.86 | [M+H]^+^ | 796.586 |
| 22 | PE 40:5 | 23.01 | [M+H]^+^ | 794.570 |
| 23 | PE 40:6 | 21.39 | [M+H]^+^ | 792.554 |
| 24 | PE 40:7 | 19.45 | [M+H]^+^ | 790.539 |
| 25 | PE 40:10 | 17.96 | [M+H]^+^ | 784.492 |
| 26 | PE 42:6 | 22.64 | [M+H]^+^ | 820.586 |
| 27 | PE 42:7 | 20.49 | [M+H]^+^ | 818.570 |
| 28 | PE 42:10 | 19.45 | [M+H]^+^ | 812.523 |

**Table S9. List of identified lipids in plasmenyl-phosphatidylcholines**

| No. | Identity | Retention time (min) | Adduct ion | Quantification ion (m/z) |
| --- | --- | --- | --- | --- |
| 1 | Plasmenyl-PC 30:0 | 20.52 | [M+H]^+^ | 690.544 |
| 2 | Plasmenyl-PC 32:0 | 21.29 | [M+H]^+^ | 718.575 |
| 3 | Plasmenyl-PC 34:0 | 23.50 | [M+H]^+^ | 746.606 |
| 4 | Plasmenyl-PC 34:1 | 22.56 | [M+H]^+^ | 744.591 |
| 5 | Plasmenyl-PC 36:0 | 26.21 | [M+H]^+^ | 774.638 |
| 6 | Plasmenyl-PC 36:1 | 24.79 | [M+H]^+^ | 772.622 |
| 7 | Plasmenyl-PC 36:2 | 22.81 | [M+H]^+^ | 770.606 |
| 8 | Plasmenyl-PC 36:3 | 20.95 | [M+H]^+^ | 768.591 |
| 9 | Plasmenyl-PC 36:4 | 20.13 | [M+H]^+^ | 766.575 |
| 10 | Plasmenyl-PC 36:5 | 20.67 | [M+H]^+^ | 764.559 |
| 11 | Plasmenyl-PC 38:1 | 27.85 | [M+H]^+^ | 800.653 |
| 12 | Plasmenyl-PC 38:3 | 22.56 | [M+H]^+^ | 796.622 |
| 13 | Plasmenyl-PC 38:4 | 20.82 | [M+H]^+^ | 794.606 |
| 14 | Plasmenyl-PC 38:5 | 20.77 | [M+H]^+^ | 792.591 |
| 15 | Plasmenyl-PC 40:1 | 30.26 | [M+H]^+^ | 828.685 |
| 16 | Plasmenyl-PC 40:2 | 27.32 | [M+H]^+^ | 826.669 |
| 17 | Plasmenyl-PC 40:4 | 27.85 | [M+H]^+^ | 822.638 |
| 18 | Plasmenyl-PC 42:5 | 27.32 | [M+H]^+^ | 848.653 |

**Table S10. List of identified lipids in plasmenyl-phosphatidylethanolamines**

| No. | Identity | Retention time (min) | Adduct ion | Quantification ion (m/z) |
| --- | --- | --- | --- | --- |
| 1 | Plasmenyl-PE 32:0 | 24.08 | [M+H]^+^ | 676.528 |
| 2 | Plasmenyl-PE 32:1 | 21.55 | [M+H]^+^ | 674.512 |
| 3 | Plasmenyl-PE 34:0 | 26.77 | [M+H]^+^ | 704.559 |
| 4 | Plasmenyl-PE 34:1 | 23.92 | [M+H]^+^ | 702.544 |
| 5 | Plasmenyl-PE 36:0 | 27.08 | [M+H]^+^ | 732.591 |
| 6 | Plasmenyl-PE 36:1 | 26.55 | [M+H]^+^ | 730.575 |
| 7 | Plasmenyl-PE 36:2 | 23.78 | [M+H]^+^ | 728.559 |
| 8 | Plasmenyl-PE 36:3 | 22.97 | [M+H]^+^ | 726.544 |
| 9 | Plasmenyl-PE 36:4 | 21.17 | [M+H]^+^ | 724.528 |
| 10 | Plasmenyl-PE 38:1 | 29.15 | [M+H]^+^ | 758.606 |
| 11 | Plasmenyl-PE 38:2 | 25.71 | [M+H]^+^ | 756.591 |
| 12 | Plasmenyl-PE 38:3 | 25.48 | [M+H]^+^ | 754.575 |
| 13 | Plasmenyl-PE 38:4 | 23.38 | [M+H]^+^ | 752.559 |
| 14 | Plasmenyl-PE 38:5 | 21.07 | [M+H]^+^ | 750.544 |
| 15 | Plasmenyl-PE 38:6 | 20.34 | [M+H]^+^ | 748.528 |
| 16 | Plasmenyl-PE 40:1 | 30.96 | [M+H]^+^ | 786.638 |
| 17 | Plasmenyl-PE 40:3 | 26.87 | [M+H]^+^ | 782.606 |
| 18 | Plasmenyl-PE 40:4 | 24.58 | [M+H]^+^ | 780.591 |
| 19 | Plasmenyl-PE 40:5 | 23.21 | [M+H]^+^ | 778.575 |
| 20 | Plasmenyl-PE 40:6 | 22.39 | [M+H]^+^ | 776.559 |
| 21 | Plasmenyl-PE 42:2 | 30.09 | [M+H]^+^ | 812.653 |
| 22 | Plasmenyl-PE 42:3 | 27.93 | [M+H]^+^ | 810.638 |
| 23 | Plasmenyl-PE 42:4 | 26.70 | [M+H]^+^ | 808.622 |
| 24 | Plasmenyl-PE 42:5 | 25.66 | [M+H]^+^ | 806.606 |
| 25 | Plasmenyl-PE 42:6 | 23.02 | [M+H]^+^ | 804.591 |

**Table S11. List of identified lipids in lysophospholipids**

| No. | Identity | Retention time (min) | Adduct ion | Quantification ion (m/z) |
| --- | --- | --- | --- | --- |
| 1 | LysoPC 16:0 | 12.40 | [M+H]^+^ | 496.340 |
| 2 | LysoPC 18:0 | 14.19 | [M+H]^+^ | 524.372 |
| 3 | LysoPC 18:1 | 12.65 | [M+H]^+^ | 522.356 |
| 4 | LysoPC 18:3 | 12.40 | [M+H]^+^ | 518.325 |
| 5 | LysoPC 20:0 | 15.46 | [M+H]^+^ | 552.403 |
| 6 | LysoPC 20:4 | 12.32 | [M+H]^+^ | 544.340 |
| 7 | LysoPC 22:0 | 13.97 | [M+H]^+^ | 580.434 |
| 8 | LysoPE 16:0 | 12.67 | [M+H]^+^ | 454.293 |
| 9 | LysoPE 18:0 | 14.39 | [M+H]^+^ | 482.325 |
| 10 | LysoPE 18:1 | 12.92 | [M+H]^+^ | 480.309 |

**Table S12. Statistical analysis results of lipids (VIP value > 1.00)**

| Name | VIP value | p(corr)[1] | *p*-value |
| --- | --- | --- | --- |
| PC 38:4 | 2.65 | 0.99 | 7.1x10^-5^ |
| PE 40:5 | 2.63 | 0.99 | 6.0x10^-4^ |
| PlsPC 38:4 | 2.61 | -0.98 | 1.2x10^-3^ |
| PlsPC 38:3 | 2.60 | -0.97 | 1.1x10^-3^ |
| PE 36:4 | 2.56 | 0.96 | 2.7x10^-3^ |
| PlsPE 40:6 | 2.53 | 0.95 | 4.0x10^-3^ |
| PE 40:6 | 2.53 | 0.95 | 4.6x10^-3^ |
| PlsPE 38:6 | 2.35 | -0.88 | 4.9x10^-2^ |
| TG 52:3 | 2.19 | 0.82 | - |
| DG 30:0 | 2.18 | 0.82 | - |
| DG 44:9 | 2.16 | 0.81 | - |
| PlsPE 42:2 | 2.06 | -0.77 | - |
| PE 34:1 | 2.05 | 0.77 | - |
| PC 32:3 | 1.97 | -0.74 | - |
| DG 32:0 | 1.91 | 0.71 | - |
| TG 52:5 | 1.82 | 0.68 | - |
| PC 42:1 | 1.80 | 0.67 | - |
| PC 42:5 | 1.78 | 0.67 | - |
| PE 34:0 | 1.72 | 0.64 | - |
| PC 38:5 | 1.69 | -0.63 | - |
| PC 40:1 | 1.56 | 0.59 | - |
| SM 38:0 | 1.55 | -0.58 | - |
| TG 46:1 | 1.55 | -0.58 | - |
| TG 46:2 | 1.52 | -0.57 | - |
| TG 58:5 | 1.51 | -0.57 | - |
| PC 38:6 | 1.46 | 0.55 | - |
| PlsPE 42:6 | 1.45 | -0.54 | - |
| PlsPE 32:0 | 1.41 | 0.53 | - |
| PC 42:2 | 1.41 | 0.53 | - |
| PlsPC 40:1 | 1.34 | 0.50 | - |
| PC 34:2 | 1.30 | - | - |
| TG 50:3 | 1.28 | - | - |
| Cer 42:1;O2 | 1.27 | - | - |
| SM 40:2 | 1.26 | - | - |
| PE 36:2 | 1.25 | - | - |
| PE 42:10 | 1.25 | - | - |
| PC 40:5 | 1.24 | - | - |
| PlsPC 32:0 | 1.23 | - | - |
| PE 42:7 | 1.22 | - | - |
| PlsPC 36:3 | 1.21 | - | - |
| TG 52:4 | 1.20 | - | - |
| TG 52:2 | 1.20 | - | - |
| PlsPE 34:0 | 1.19 | - | - |
| PE 32:1 | 1.18 | - | - |
| PC 42:6 | 1.17 | - | - |
| SM 34:1 | 1.15 | - | - |
| TG 56:1 | 1.11 | - | - |
| DG 34:1 | 1.10 | - | - |
| PlsPE 40:4 | 1.09 | - | - |
| PlsPC 30:0 | 1.09 | - | - |
| PC 38:3 | 1.09 | - | - |
| LPC 22:0 | 1.06 | - | - |
| SM 40:0 | 1.06 | - | - |
| PE 32:0 | 1.05 | - | - |
| TG 44:1 | 1.04 | - | - |
| PlsPC 38:5 | 1.01 | - | - |
| PE 38:5 | 1.01 | - | - |
| LPE 16:0 | 1.01 | - | - |
| PlsPC 34:1 | 1.01 | - | - |

**Table S13. Ten individual GA runs molecular docking simulation information table, E = binding energy (kcal/mol); KI = inhibition constant (mM)**

| No. | Pcyt2 | | Selenoi | |
| --- | --- | --- | --- | --- |
|  | E | KI | E | KI |
| 1 | -4.35 | 0.65 | -4.36 | 0.64 |
| 2 | -4.07 | 1.04 | -4.01 | 1.15 |
| 3 | -3.59 | 2.33 | -3.76 | 1.76 |
| 4 | -2.90 | 7.54 | -3.43 | 3.08 |
| 5 | -2.84 | 8.35 | -3.27 | 4.00 |
| 6 | -2.72 | 10.08 | -2.66 | 11.20 |
| 7 | -2.51 | 14.45 | -2.49 | 15.03 |
| 8 | -2.39 | 17.62 | -2.36 | 18.63 |
| 9 | -2.34 | 19.11 | -2.29 | 20.81 |
| 10 | -2.24 | 22.90 | -0.70 | 306.65 |
